# Environmental impact of potentially toxic elements on soils, sediments, waters, and air nearby an abandoned Hg-rich fahlore mine (Mt. Avanza, Carnic Alps, NE Italy)

**DOI:** 10.1007/s11356-023-26629-7

**Published:** 2023-04-14

**Authors:** Nicolò Barago, Cristiano Mastroianni, Elena Pavoni, Federico Floreani, Filippo Parisi, Davide Lenaz, Stefano Covelli

**Affiliations:** Dipartimento di Matematica e Geoscienze, Università di Trieste, Via Weiss 2, 34128 Trieste, Italy

**Keywords:** Tetrahedrite-tennantite, Contamination, Potentially toxic trace elements, Gaseous elemental mercury (GEM), Decommissioned mines, Mercury (Hg), Antimony (Sb), Arsenic (As)

## Abstract

The decommissioned fahlore Cu-Sb(-Ag) mine at Mt. Avanza (Carnic Alps, Italy) is a rare example of exploited ore deposits, as the tetrahedrite (Cu_6_[Cu_4_(Fe,Zn)_2_]Sb_4_S_13_) is the main ore mineral found. This multi-compartmental geochemical characterisation approach provides one of the first case studies regarding the geochemical behaviour and fate of Hg, Sb, As, Cu, and other elements in solid and water matrices and of Hg in the atmosphere in an environment affected by the mining activity of a fahlore ore deposit. Elevated concentrations of the elements (Cu, Sb, As, Pb, Zn, Hg) associated with both (Zn-Hg)-tetrahedrite and to other minor ore minerals in mine wastes, soils, and stream sediments were observed. Concentrations in waters and stream sediments greatly decreased with increasing distance from the mining area and the I_geo_ index values testify the highest levels of sediment contamination inside the mine area. Thallium and Ge were associated with the “lithogenic component” and not to sulfosalt/sulphide minerals. Although mine drainage water often slightly exceeded the national regulatory limits for Sb and As, with Sb being more mobile than As, the relatively low dissolved concentrations indicate a moderate stability of the tetrahedrite. The fate of Hg at the investigated fahlore mining district appeared similar to cinnabar mining sites around the world. Weak solubility but the potential evasion of gaseous elemental mercury (GEM) into the atmosphere also appear to be characteristics of Hg in fahlore ores. Although GEM concentrations are such that they do not present a pressing concern, real-time field surveys allowed for the easy identification of Hg sources, proving to be an effective, suitable high-resolution indirect approach for optimising soil sampling surveys and detecting mine wastes and mine adits.

**Supplementary Information:**

The online version contains supplementary material available at 10.1007/s11356-023-26629-7.

## Introduction

The spread of potentially toxic elements (PTEs) in environmental compartments has often been closely associated with the mining industry, in particular with the extraction and processing of a variety of metal(loid)-bearing sulphide minerals (e.g. Higueras et al. 2006). Similar to mining districts that are still in operation, decommissioned mines may remain a potential source of PTEs which may be released into the environment for decades or even centuries. The recovery of metal(loid)s from ores involves extraction, crushing, milling, and different separation processes, e.g. roasting in the case of Hg or froth flotation in the case fo Zn and Pb. In the vicinity of ore dressing and smelting plants, the release of contaminated waste in solid and liquid forms, leaching of PTEs from mine waste (Barago et al. 2023), or the escape of fumes containing metal(loid)s vapour and dust (Kotnik et al. 2005) following the dry and wet deposition of the element may be responsible for widespread contamination of the environment (Gosar and Teršič 2012). Additionally, eroded mine waste particles can be mechanically removed by flood events and accumulated in riverbank deposits downstream from the mine (Gosar et al. 1997), representing a secondary source of contamination even at considerable distances from mining sites.

Mercury (Hg), antimony (Sb), and arsenic (As) are often considered priority contaminants due to their potentially toxic effects (DFG 1994; US EPA 1999; UNEP 2019). Centuries of anthropogenic activities, such as mining and burning fossil fuels, have contributed and still contribute in large part to a significant increase in the total amount of Hg and Sb emissions into the environment (Smichowski 2008; Selin 2009). In fact, only a small fraction (< 5%) of these emissions are associated with primary geogenic sources (i.e. volcanoes, geothermal activities) and weathering of naturally enriched rocks and soils (Hinkley et al. 1999; Shotyk et al. 2004; Driscoll et al. 2013), whereas the remaining portion is imputed to primary anthropogenic inputs and secondary re-emissions (Driscoll et al. 2013; UNEP 2019).

However, different PTEs are characterised by different behaviour and fate, and consequently mobility and bioavailability. Some PTE-bearing sulphides such as cinnabar (HgS), being resistant to normal oxidation and physico-chemical alteration processes, are extremely insoluble in water and can enter the hydrogeochemical cycle through abiotic transport pathways mainly in the form of mechanically degraded solid particles (Biester et al. 2000; Covelli et al. 2007) rather than in the dissolved phase (Gray et al. 2004; Li et al. 2012). In some cases, such as world-class Hg mine sites (e.g. Almadén, Idrija, Monte Amiata) (Esbrí et al. 2010), riverine water draining the mining districts can also be enriched in dissolved Hg (Kocman et al. 2011). However, since Hg is a generally poorly soluble metal, it is more often associated with suspended particulate matter (Baptista-Salazar et al. 2017), colloidal fraction (Lowry et al. 2004), and stream sediments (Gosar et al. 1997). The element dispersion in the entire river basin downstream from the mining area (e.g. Chiarantini et al. 2016; Hines et al. 2000; Garcia-Ordiales et al. 2017, 2019; Gray et al. 2014, 2015) is the result of runoff or drainage of mine waste, including the calcines produced during roasting of the ore (Rytuba 2000). Mercury in particular, being a volatile element, can be released to the atmosphere in gaseous elemental mercury (GEM) form from surfaces where Hg can already be present in the substrate as native Hg, as a primary mineral, together with the sulphide (cinnabar) (Higueras et al. 2012; Loredo et al., 2007), or as a by-product of ore processing (Gray et al., 2010; Kotnik et al. 2005). Moreover, the evasion of GEM usually derives from the reduction of Hg^2+^ forms to Hg^0^ through both abiotic and biotic pathways mainly controlled by solar radiation, and air and soil temperatures (Choi and Holsen 2009; Wang et al. 2005). The primary control over GEM evasion into the atmosphere is exerted by the presence of volatile Hg compounds and Hg concentrations in the substrate. Hence, high volumes of Hg-bearing minerals in Hg mining areas usually lead to the notable stimulation of Hg emission (Agnan et al., 2016). Furthermore, sunlight and heat promote Hg volatilisation (Carmona et al. 2013)

Arsenic and Sb are metalloids and are either associated often together in sulphide ores or related to anthropogenic sources (Filella et al. 2002a). The common primary sulphide minerals of As include arsenopyrite (FeAsS), orpiment (As_2_S_3_), and realgar (AsS); of Sb is stibnite (Sb_2_S_3_). The predominant forms of As and Sb in nature are the +3 and +5 oxidation states, mainly found in reducing and oxidising environments, respectively. Geochemical triggers to As mobilisation in waters may be different such as (1) desorption at high pH under oxic conditions and (2) reducing environment (Hounslow 1980; Smedley and Kinniburgh 2002). However, in near-neutral to slightly alkaline environments, As may not be mobile (Barago et al. 2023); in contrast, Sb appears to be more mobile with respect to As (Majzlan et al. 2018).

In addition to cinnabar, which is the most widespread Hg-bearing mineral, and therefore the most commercially exploited for the production of elemental Hg all over the world, there are about twenty minerals in nature, mainly sulphur compounds in association with Zn, Fe, and other metals, containing variable amounts of Hg. Mercury is found as a native metal only in small quantities. Fahlore minerals from the tetrahedrite-tennantite group, which are Cu-Sb and Cu-As sulfosalts, respectively, are abundant in many types of mineral deposits (Ciobanu et al. 2005; Apopei et al. 2016; Lyubimtseva et al. 2019). They have recently been of interest to the scientific community for their high compositional variability and historic importance for Cu and Ag extraction and, other trace elements (Hg, Bi, Te, Cd, Pb, Se) (Johnson et al. 1986; Sack and Ebel 1993; Karup-Møller and Makovicky 2003). Although the tetrahedrite mineral group has already been recognised as a contamination source of As and Sb (Borčinová Radková et al. 2017) along with Hg in some cases, very little is known about what is left over from the extraction activity of fahlore mineralisations.

The occurrence of potentially toxic elements (PTEs), with a particular focus on As, Hg, Sb, in several environmental matrices from active and decommissioned mining sites characterised by “non-traditional” (e.g. cinnabar, stibnite) ore deposits represents an issue of environmental concern. A growing number of recent and on-going studies have examined the characteristics of (Cu, Sb, As)-rich minerals from the tetrahedrite-tennantite group as source of contaminants, the geochemical behaviour of PTEs at such mining sites, and the subsequent environmental issues related to PTE mobility (e.g. Borčinová Radková et al. 2017; Higueras et al. 2012; Majzlan et al. 2018). However, little information is currently available regarding the role of such minerals in terms of the release of PTEs into riverine water and the atmosphere. New studies can provide more information with which to establish cause-and-effect linkages among the geological attributes and the environmental behaviour, further developing geoenvironmental models for fahlore ore deposits (Plumlee et al., 1999; Seal II and Foley, 2002).

The main purposes of this research aim at assessing the environmental impact and PTE behaviour in soils, stream sediments, mine drainage and surface waters, and air around one of the historical Cu-Sb fahlore ore deposits in Europe, which is a rare example of exploited ore deposit as the tetrahedrite is the main ore mineral found.

## Material and methods

### Geological setting

**Fig. 1:**
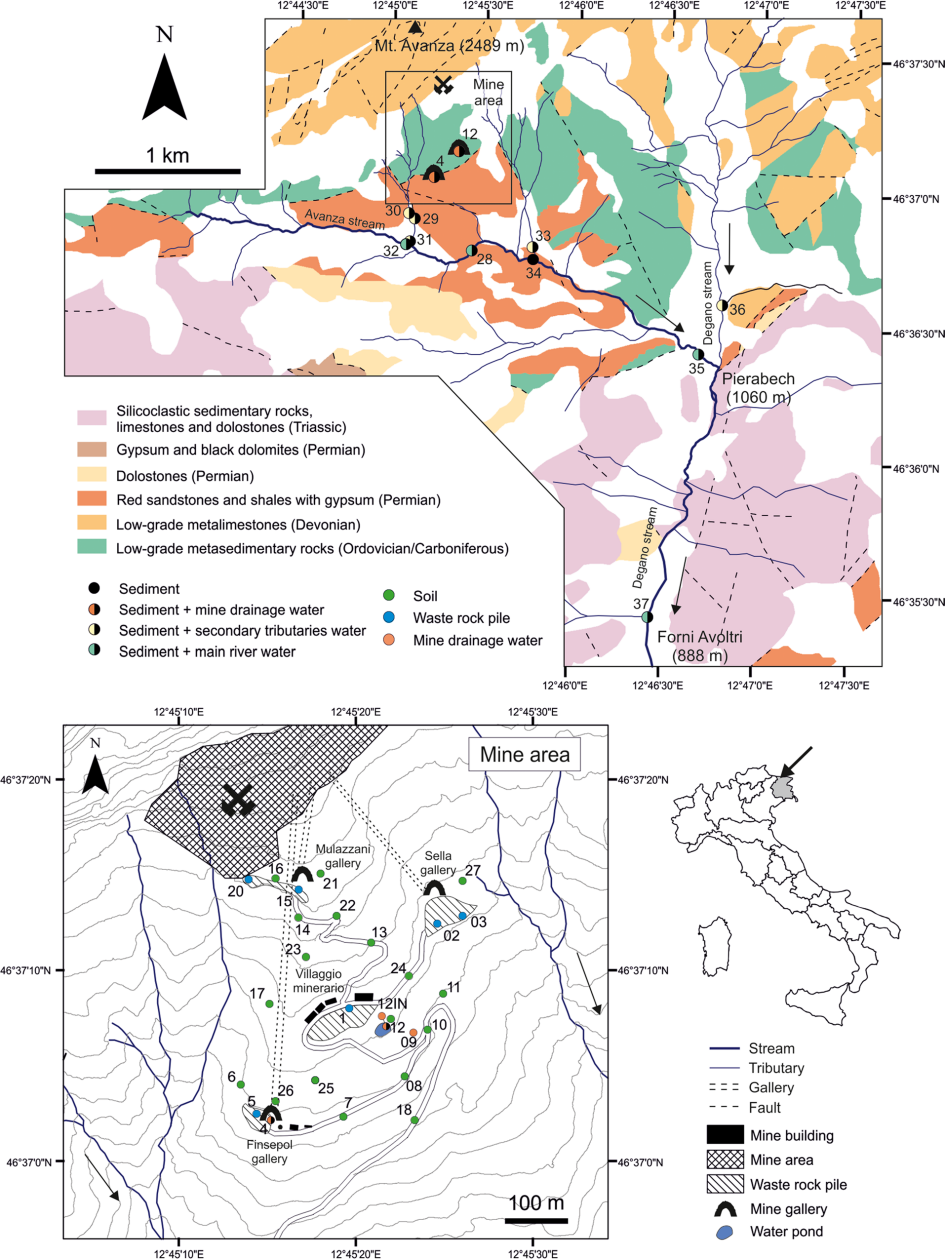
Geological sketch of the Mt. Avanza mining district with location of the sampling sites. Geological units from Venturini et al. (2001)

The carbonate-hosted stratabound, siliceous crust type (SCT), fahlore ore deposit of Mt. Avanza (Brigo et al. 2001) belongs to the Palaeocarnic Devonian-Lower Carboniferous (Dinantian) metallogenic province, whose mineralisation is bound to a palaeorelief of Devonian limestones transgressively overlain by Lower Carboniferous to Lower Permian clastic sediments in the Palaeocarnic Chain (Spalletta et al. 2021, and references therein, Fig. 1). It is thought that they are genetically associated with metal-bearing hydrothermal fluids linked to the Middle Carboniferous magmatic event (Brigo et al. 1988). The mineralisation crops out for 100 km in the Carnic Alps (Italy and Austria) and 50 km in the Karawanken (Slovenia) to the east, evidence of a regional event. The province is highly variable, being characterised by copper-dominant fahlore (tetrahedrite), base metal sulphides, barite, and fluorite, with predominating Hg-Cu in the west, Zn-Sb-Cu and Zn alone in the centre, and Ba in the east of the Palaeocarnic Chain (Brigo et al. 1988, 2001).

The Palaeocarnic Chain, which is the northernmost east-west–oriented sector of the Friuli Venezia Giulia Region consists of a sequence characterised, from W to E, by metamorphic rocks of the greenschist facies to mainly sedimentary rocks overlying the crystalline basement (Brime et al. 2008), with the oldest units belonging to the Ordovician. The area is directly south of the dextral transpressive Gail Line, which is part of the Periadriatic Fault system delineating the boundary between the European and Adriatic plates (Doglioni 1988; Schmid et al. 1989; Handy et al. 2015).

The fahlore Cu-Sb(-Ag) Mt. Avanza mineralisation is hosted in a sub-vertical south-dipping tectonic contact between the Devonian limestones which are represented by the Mt. Avanza and Mt. Navastolt reliefs, and deformed low-grade metasedimentary rocks, the age of which are still subject to debate (Carboniferous or Ordovician) (Di Colbertaldo 1960; Feruglio 1966; Spalletta et al. 1981; Venturini et al. 2001; Venturini 2006). Such low-grade metamorphic metasedimentary rocks (phyllites) alternating with quartzite beds possibly belong to the Val Visdende and Fleons Formations (Ordovician) mixed with scanty tectonic slices of low-grade metamorphic turbidites of the Hochwipfel Formations (Carboniferous) (Venturini et al. 2001). At the foot of the Mt. Avanza mining area, Permian red sandstones from the Val Gardena Formation crop out, whereas in the area surrounding the Avanza Valley, Permian gypsums and dolostones pertaining to the Bellerophon Formation are reported.

#### The mineral assemblage

The paragenesis is constituted by sulfosalts and sulphides where massive and microcrystalline tetrahedrite is the most abundant mineral phase with minor galena. Limited occurrence of sphalerite is reported together with rare to very rare pyrite, chalcopyrite, and cinnabar (Bortolozzi et al. 2015; Ciriotti et al. 2006; Di Colbertaldo 1960; Dondi et al. 1995; Feruglio 1966; Pirri 1977). The chemical composition of tetrahedrite was found to be variable and generally enriched in Zn and/or Hg, with Zn contents ranging from 4.01 to 6.97 wt.% and Hg from 1.48 to 8.66 wt.% (Table 1; Casari 1996).

**Table 1:** Electron microprobe analyses of tetrahedrite from Mt. Avanza (wt. %) (Casari 1996)

	Cu	Ag	Hg	Fe	Zn	Sb	As	S
Average	37.6	0.20	4.26	0.37	5.62	23.9	3.69	24.9
Min	35.5	0.10	1.48	0.09	4.01	20.5	1.50	23.6
Max	39.4	0.29	8.66	1.01	6.97	27.9	5.66	25.7

The tetrahedrite group has recently been defined as represented by five different series on the basis of the constituents (Biagioni et al. 2020a). Tetrahedrite is a complex sulfosalt with a general formula Cu_6_[Cu_4_(Fe,Zn)_2_]Sb_4_S_13_ which can host many minor components in its lattice structure (such as As, Ag, Hg, Cd, Mn, Bi, Te, Se). In particular, Hg can be present in variable amounts, and when the Hg content is extremely high in tetrahedrite (up to 22.70 wt.%) it can be referred to tetrahedrite-(Hg), by using the general formula: Cu_6_(Cu_4_Hg_2_)Sb_4_S_13_ (Biagioni et al. 2020b). Tetrahedrite with variable Hg content can be found in several kinds of deposits around the world: (1) cinnabar deposits, (2) Hg-Sb deposits, (3) Hg tetrahedrite-tennantite deposits, and (4) low-Hg tetrahedrites in Sb-W, Pb-Ag, and Au deposits (Mozgova et al. 1979).

Casari (1996) reported that two types of tetrahedrite were found at Mt. Avanza: a Hg-Agrich and a Zn-Ag-rich member. The tetrahedrite specimen with the highest concentrations of Hg is represented by the following compositional formula:

$${\left(C{u}_{9.91}A{g}_{0.06}F{e}_{0.13}Z{n}_{1.24}H{g}_{0.55}\right)}_{11.89}{\left(S{b}_{3.17}A{s}_{0.92}\right)}_{4.09}{S}_{13.02}$$

In different horizons, mixed varieties of tetrahedrite-tennantite with a Sb:As ratio of 1:1 can occasionally be found. Copper carbonates secondary minerals are very frequent (e.g. azurite, malachite), due to the contact with carbonate water, in association with secondary Sb-minerals (so-called antimony ochre), minor Fe-oxy-hydroxides and Pb secondary carbonate minerals (cerussite). The presence of Sb or As supergene minerals generally may depend on the amount of tetrahedrite or subordinates tennantite phases, respectively, that has undergone alteration (Dondi et al., 1995). Such Sb-bearing secondary minerals possibly belong to the Roméite group (Bortolozzi et al. 2015). The main non-metallic and gangue minerals are baryte and quartz, followed by calcite and dolomite (Feruglio 1966).

Due to the sub-vertical distribution of the mineralisation, mine galleries have been realised over the years at different levels to reach the mineralised tectonic contact. The following galleries from N to S can be found: Bauer (1861 m a.s.l.), O’Conor (1815 m a.s.l.), Mulazzani (1780 m a.s.l.), Q. Sella (1686 m a.s.l.) and Finsepol (1640 m a.s.l.) (Fig. 1).

### Study area

**Fig. 2:**
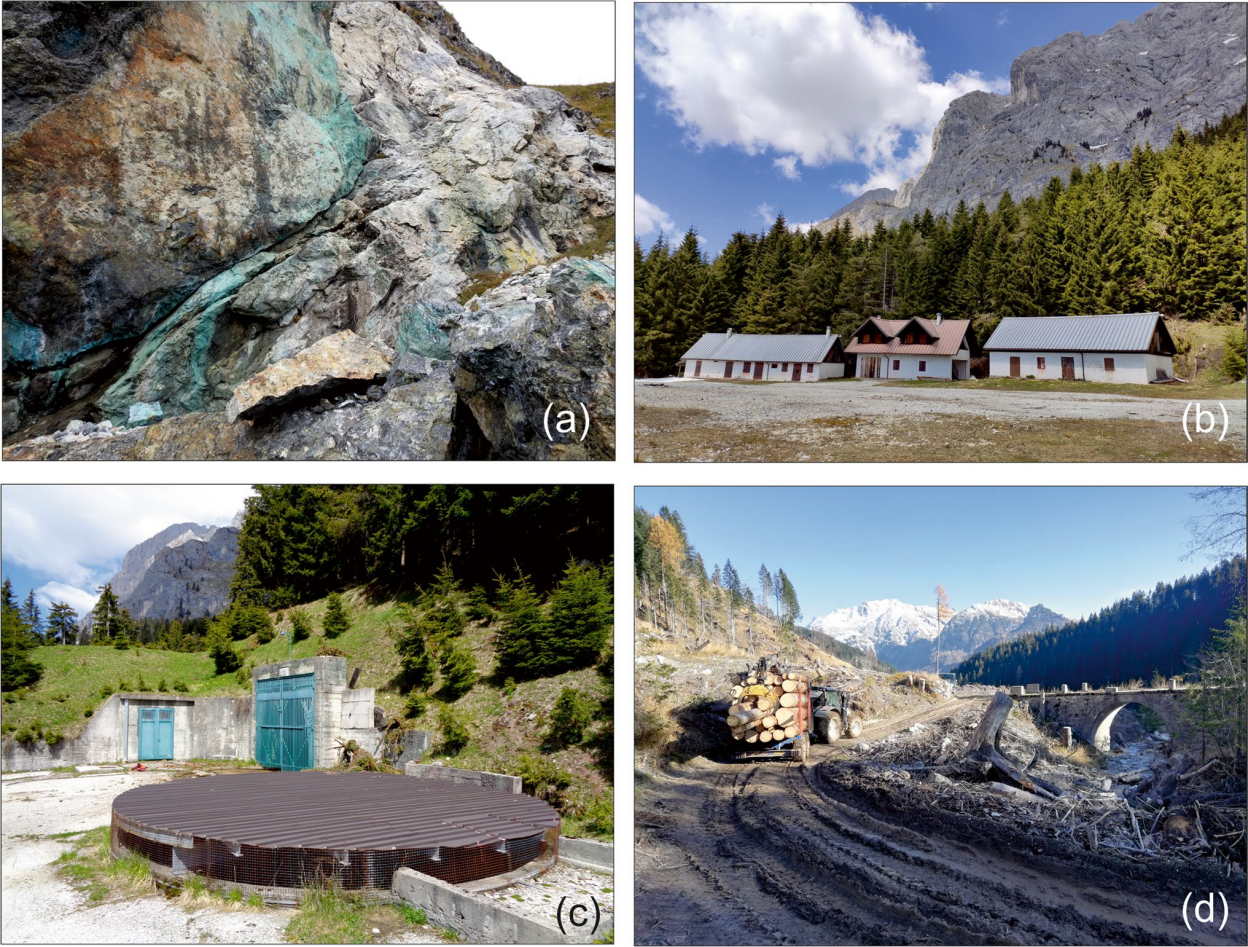
**a** Outcrop of alteration supergene minerals related to the tetrahedrite Cu-Sb-(Ag) mineralisation “Pietra Verde”, **b** mining village of the Mt. Avanza mine (“Villaggio minerario”), **c** Finsepol gallery, **d** Avanza valley and Avanza stream

The study area is located between 900 and 1900 m a.s.l., in the Avanza Valley (Fig. 2d) (Carnic Alps, Friuli Venezia Giulia, Italy) near the village of Forni Avoltri, on the border with Austria and the Region of Veneto. The landscape is characterised by an Alpine mountainous environment morphologically controlled by past glacial and present fluvial erosion and covered by coniferous forest.

The Cu-Sb-(Ag) Mt. Avanza mine was an important Cu ore deposits in the Eastern Alps during the nineteenth century (Feruglio 1966) and is historically important as it might have been active since the European Bronze Age (Artioli et al. 2020). A comprehensive review of the history of the Mt. Avanza mining site is reported in Zucchini (1998). The first document mentioning the mine dates back to 778 AD. The mine was in operation intermittently until 1952, when extraction definitively ended. The last phase of mineral exploration began in 1975, as evidenced by the realisation of the “Finsepol gallery” (Fig. 2c) and the largest waste rock pile near the mining village “Villaggio Minerario” (Fig. 2b), but no recovery of Cu-Ag followed, and exploration permanently halted in 1995. The area has been abandoned since then. Until the end of the nineteenth century, metals were recovered using roasting processes in Pierabech, near the village of Forni Avoltri (Fig. 1), while a flotation plant was operating for a couple of years during World War II (Feruglio 1966).

### Sampling strategy

Sampling campaigns in the Mt. Avanza decommissioned mining district were performed in June, September, October, and November 2021. Solid (ore-bearing rocks, soils, mine wastes, sediments) and water (mine drainages, main river and secondary tributaries) samples were collected, whereas atmospheric GEM was monitored in the field.

After removing organic debris (e.g., roots, leaves), almost 1 kg of a composite sample of the most surficial layer (0–10 cm) of soils and mine waste piles was collected at each selected site (Fig. 1). Sediments were collected immediately downstream from the mine drainages (MA4 and MA12 sites) and along the main stream (Rio Avanza) and its tributaries. Ore-bearing rocks of > 5 cm in diameter and characterised by noticeable ore minerals were collected in the mine site near the main waste piles.

The physico-chemical parameters of water (temperature, pH, redox potential (ORP), electrical conductivity (EC), dissolved oxygen (DO) and total dissolved solids (TDS)) were measured in situ using a portable probe (Hanna HI98194). All the collected water samples and field blanks prepared with ultrapure water were preventively filtered in situ (syringe filters Millipore Millex HA, 0.45 μm) and separated in different aliquots for distinct chemical analysis. Water aliquots for PTE and major cation analytical determinations were stored in HDPE vessels and acidified with HNO_3_ (1% v/v, HNO_3_ 67-69% v/v VWR), whereas water aliquots for Hg determination were collected in borosilicate glass containers and immediately oxidised with bromine chloride (BrCl, 0.5% v/v).

### Chemical elemental analysis of the solid matrix

Solid samples were first air-dried in the laboratory at a temperature of 25 °C to minimise the loss of Hg due to its easy volatilisation. Then, each dried sample was sieved at 2 mm and finely ground in tungsten carbide mills.

For the determination of major and trace elements, with the exception of Hg, aliquots were acid-digested in PTFE vessels through a total dissolution in a closed microwave system (Multiwave PRO, Anton Paar) using inverse aqua regia (HNO_3_ 67–69% v/v and HCl 34-37% v/v, VWR, 3:1), HF (47–51% v/v, VWR), and H_2_O_2_ (30% w/v) according to US EPA method 3052 (US EPA 1996). Blanks were prepared for each microwave batch to check the analytical performance. The samples were subjected to two heating steps for mineralisation, and boric acid (H_3_BO_3_, 6%) was added in the second step to buffer HF excesses. The obtained solutions were diluted up to a final volume of 25 mL by adding Milli-Q water and filtered through syringe filters (Millipore Millex HA, 0.45 μm) before analytical determinations. Concentrations of major and trace elements were determined via inductively coupled plasma–mass spectrometry (ICP-MS, NexION 350X equipped with an ESI SC autosampler, PerkinElmer) using the kinetic energy discrimination (KED) mode to avoid and minimise cell-formed polyatomic ion interference. The calibration of the instrument was performed via the analysis of standard solutions prepared by dilution (ranging between 0.5 and 500 μg/L) from two multistandard solutions (Periodic Table MIX 1 e MIX 2 for ICP, TraceCERT Sigma-Aldrich) and acidified with HNO_3_ (1% v/v). Moreover, certified reference material (PACS-3 Marine Sediment Certified Reference Material, NRCC, Canada) was digested in the same batch as the solid samples to assess the accuracy of the analysis. Acceptable recoveries were obtained varying between 80 and 109%, and the precision of the analysis expressed as RSD% was < 3%.

Total Hg (THg) was determined using a direct mercury analyser (DMA-80 Milestone), in accordance with EPA method 7473 (US EPA 1998). Each sample was analysed in triplicate, and the quality of the analysis was evaluated using a certified reference material (PACS-3 Marine Sediment CRM, NRCC, Canada). The relative standard deviation of at least three determinations was < 2%.

### Mineralogical determinations of the solid matrix

Manually separated ore minerals (*n* = 8) from rock fragments sampled on the waste rock piles were grounded with an agate mortar and analysed by means of the powder XRD technique using a STOE D500 (Siemens, Monaco, Germany) diffractometer with Cu Kα radiation (λ=1.5418Å), monochromatised by a secondary flat graphite crystal. The scanning angle ranged from 5 to 90° of 2*θ*, steps were of 0.005° of 2*θ*, and the counting time was of 6 s/step. The current used was 20 mA and the voltage 40 kV. The “Match!” software version 3.14 and the reference patterns calculated from the COD (Crystallography Open Database) database were used for phase identification.

### Chemical analysis of the water matrix

The concentrations of major cations (Ca^2+^, Mg^2+^, Na^+^, and K^+^) in water samples were determined via inductively coupled plasma–optical emission spectrometry (ICP-OES) using an Optima 8000 Spectrometer (Perkin Elmer, USA) equipped with a S10 Autosampler. Instrument calibration was performed using standard solutions (ranging between 0.1 and 100 mg/L) prepared by dilution from a multistandard solution (Periodic Table MIX 5 for ICP, TraceCERT Sigma-Aldrich) and acidified with HNO_3_ (1%, v/v). The precision of the analysis expressed as RSD% was <5%. The analytical determination of major anions (F^-^, Cl^-^, NO_3_^-^, SO_4_^2-^) was performed via ion chromatography (IC, Dionex IonPac™ AS9-HC, Thermo Scientific™). The instrument was calibrated using 7 standard solutions (ranging between 0.1 and 100 mg/L) prepared by dilution from multistandard solutions (Anion multi-element standard I and Anion multi-element standard II, Merck). Each standard was analysed in triplicate. Moreover, three aliquots of the company’s calibration solutions (Dionex Seven Anion Retention Time Standard Concentration, Thermo Scientific™) were analysed for quality control. With regard to CO_3_^2-^ and HCO^3-^, the determination was made using potentiometric titration using 0.01N HCl (APAT and IRSA-CNR 2003).

The analytical determination of trace elements was performed via inductively coupled plasma–mass spectrometry (ICP-MS, NexION 360X equipped with an ESI SC autosampler, PerkinElmer), using the KED mode, and scandium (Sc), yttrium (Y), and holmium (Ho) were used as internal standards in order to evaluate and check for potential matrix effects. For the analysis of the water matrix, the instrument was calibrated using 5 standard solutions (ranging between 0.5 and 10 μg/L) prepared by dilution of two multistandard solutions (Periodic Table MIX 1 and Periodic Table MIX 2 for ICP, TraceCERT Sigma-Aldrich) and acidified with HNO_3_ (1%, v/v). The precision of the analysis expressed as RSD% was <3%.

Dissolved Hg in water samples was determined by Cold Vapor Atomic Fluorescence Spectrophotometry coupled with a gold trap pre-concentration system (CV-AFS Mercury, Analytik Jena), according to EPA method 1631e (US EPA 2002). Before the analytical determination, a pre-reduction using NH_2_OH-HCl (250 μL/100 mL sample) was performed until the yellow colour disappeared, followed by a reduction with SnCl_2_ (2% v/v in HCl 2% v/v). The instrument was calibrated using standard solutions (ranging between 1 and 50 ng/L) prepared by diluting a Hg standard solution (mercury standard solution, Merck) and acidified with BrCl (0.5%, v/v). The precision of the analysis expressed as RSD% was < 3%.

### On-site monitoring of gaseous elemental mercury (GEM)

Measurements of gaseous elemental mercury (GEM) were conducted by means of a Lumex RA-915M Hg portable analyser during a single survey in October 2021. The instrument is an atomic absorption spectrometer (AAS) with Zeeman background correction and high frequency modulation of light polarisation that provides both high sensitivity and minimal interference (Sholupov and Ganeyev 1995). The accuracy of the method is 20% and the dynamic range is 2–25,000 ng/m^3^. Values below the limit of detection (LOD = 2 ng/m^3^) were treated with the medium bound approach thus set to 50% of the LOD (US EPA 2000). Data were acquired continuously with an integration time of 10 s as average values of concentrations observed every 1 s and stored in instrument datalogger. Baseline checks were performed at the beginning and at the end of measurement session and constantly each 15 min during sampling. Monitoring was performed by car along all the access roads from Pierabech to the mine village at a constant speed of 5–10 km/h and on foot in the mining area, focusing on spatial distribution of GEM around possible point sources (e.g. mine wastes, gallery entrances). GPS coordinates were acquired in parallel with the measurements. For sampling by car, the inlet of the instrument was connected to a 1-m-long PVC tube mounted outside the vehicle window. Spatial distribution of GEM concentration was assessed with QGIS software and graphically represented using inverse distance weighting (IDW) interpolation.

## Results and discussion

### The mine area

The concentrations of PTEs in soils, mine wastes, and sediments were largely variable, from few to thousands of milligram per kilogram, but never exceeding 1%. In detail, the maximum concentrations in the mining district found were Cu = 4019 mg/kg, Sb = 1049 mg/kg, Pb = 1216 mg/kg, Zn = 1204 mg/kg, As = 654 mg/kg, and Hg = 473 mg/kg, demonstrating that ore-bearing minerals were diffused in the area as a result of mining (Table 2, Table S1).

**Table 2:** Summary of the concentrations (min-max) of the main metals and metalloids in the environmental matrices

	Type (*n* samples)	As	Cu	Hg	Sb	Pb	Zn
		*mg/kg*	*mg/kg*	*mg/kg*	*mg/kg*	*mg/kg*	*mg/kg*
**Solid**	Waste rock pile (6)	8.20-654	31.3-4019	1.42-473	5.00-1049	19.4-397	20.3-553
	Soil (18)	12.1-162	10.1-1557	0.21-132	2.74-153	26.4-1216	62.0-1204
	Sediment (12)	12.9-212	14.0-242	0.04-9.15	1.64-28.0	14.7-78.8	57.1-171
		*μg/L*	*μg/L*	*ng/L*	*μg/L*	*μg/L*	*μg/L*
**Water**	Mine drainage (11)	0.87-14.8	0.29-8.28	2.41-13.2	4.33-20.3	< LOD-1.02	< LOD-11.0
	Tributaries (5)	0.18-3.89	0.12-0.41	1.81-10.2	0.19-1.59	< LOD	< LOD-1.90
	Main river (5)	0.60-2.98	<LOD-1.08	1.17-6.49	0.30-1.16	< LOD-0.72	< LOD-4.30
				*ng/m*^*3*^			
**Gas**	Mine area (1222)	n.d.	n.d.	< LOD-25.4	n.d.	n.d.	n.d.
	Avanza valley ^a^ (344)	n.d.	n.d.	< LOD-13.7	n.d.	n.d.	n.d.
	Rural area ^b^ (134)	n.d.	n.d.	< LOD-4.50	n.d.	n.d.	n.d.

^a^Between the mine and the downstream rural area

^b^The villages of Pierabech and Forni Avoltri (Fig. 1)

**Fig. 3:**
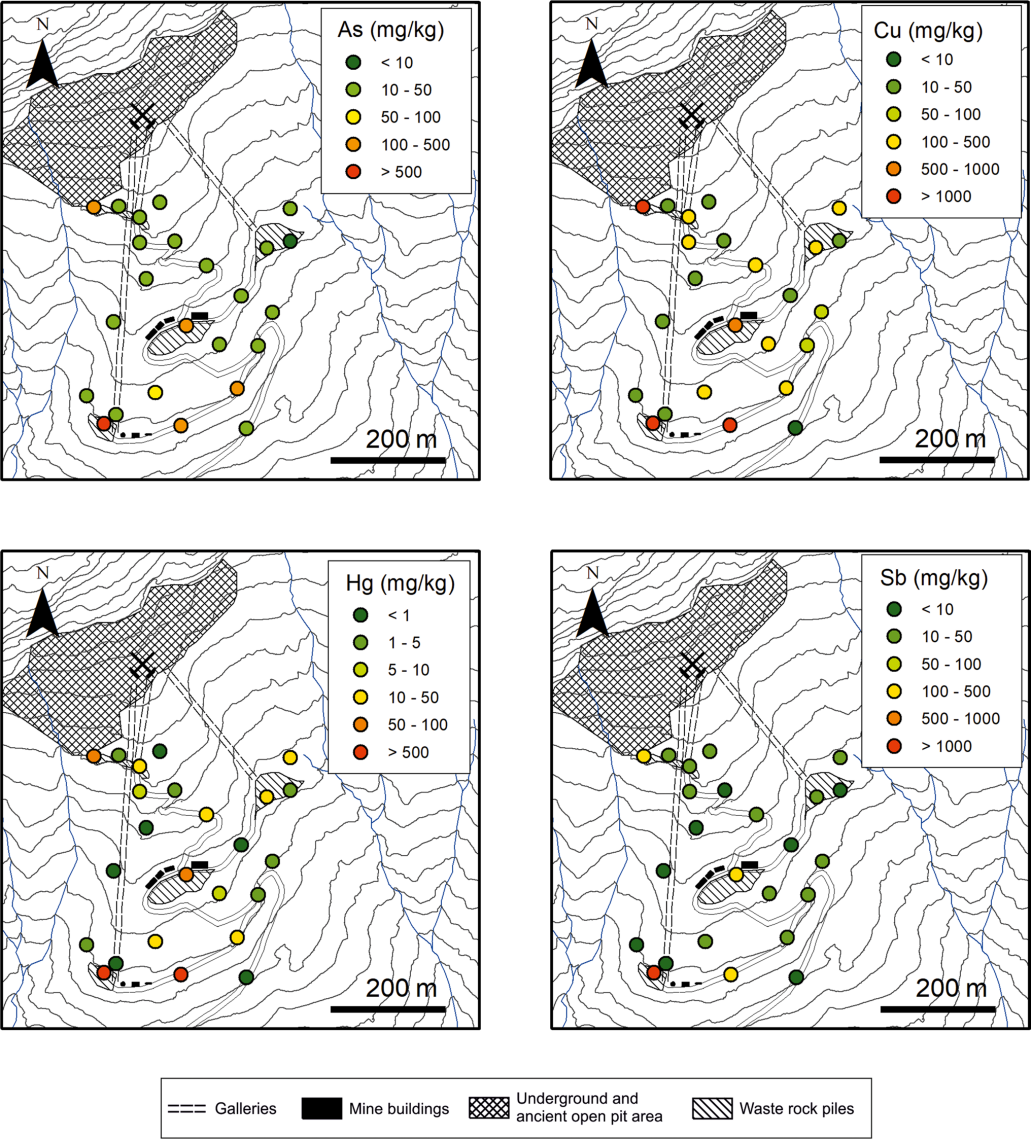
Distribution of As, Cu, Hg, and Sb concentrations in the surficial soils and waste rocks of the Mt. Avanza mining area

Whereas maximum concentrations were associated with waste rock piles, the soils around these waste deposits were also heavily enriched in PTEs (Fig. 3). Elevated concentrations (e.g. 132 mg/kg of Hg and 1557 mg/kg of Cu) can indeed be found in surficial soils due to the extensive use of by-product gravel and sands for the construction of the main access roads and mine infrastructure. However, great variability occurred depending on mining operations and the natural distribution of the different elements.

**Fig. 4:**
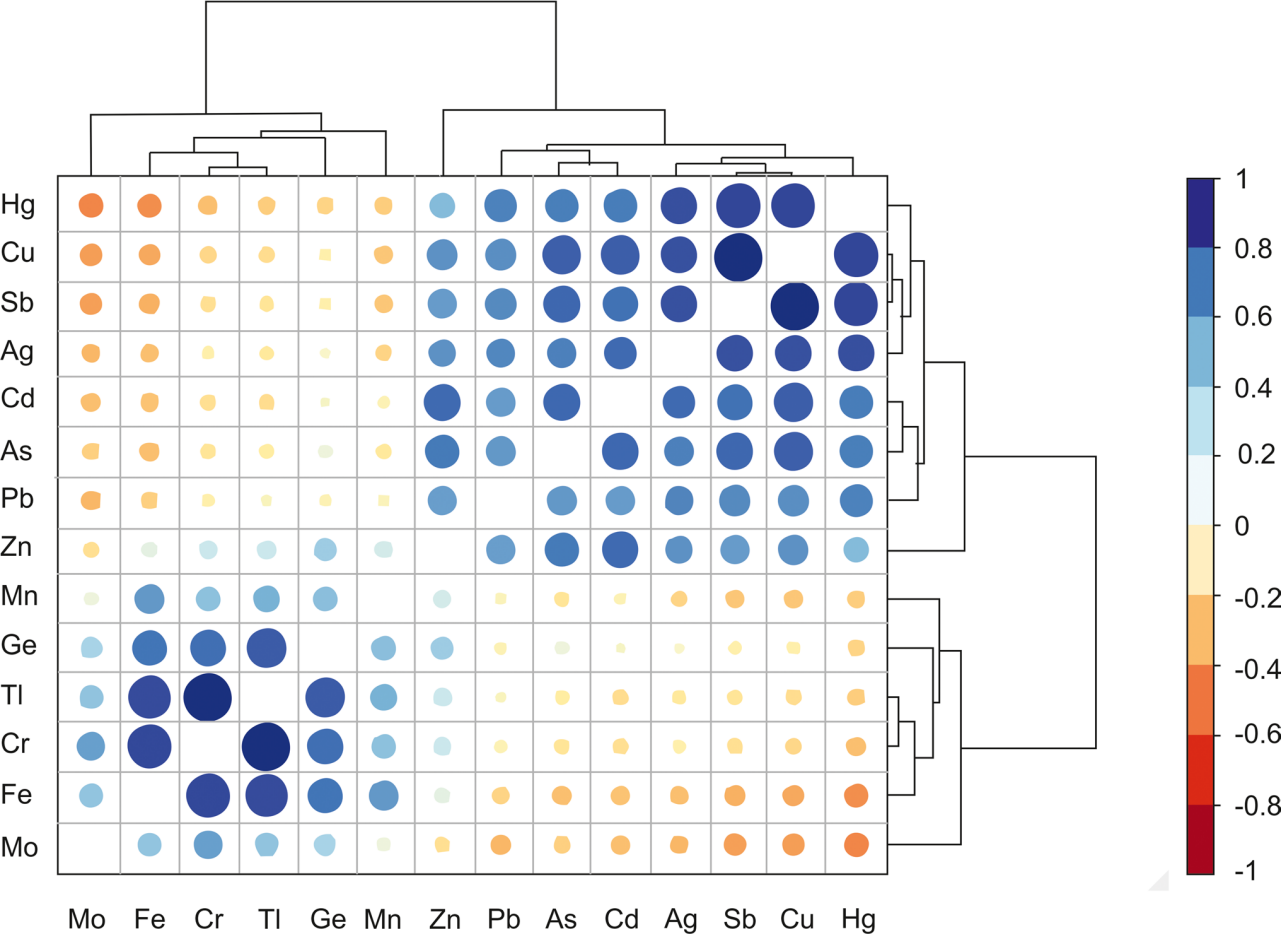
Pearson correlation heatmap of major and trace elements in all waste rocks, soils, and sediments analysed (processed using the heatmaply package; Galili et al. 2018). The colour is a function of the correlation coefficient (r), whereas point size is log function of the *p* value

Although the concentrations of metal(loid)s in soils and waste rocks are in the same order of magnitude, Pb-Zn distribution can be discordant with respect to that of Cu-Sb-Hg-As, indicating that the source of metal(loid)s (i.e. the ore minerals of the mineralised veins) was heterogeneous and composed of multiple minerals. The Cu-Sb-Hg-As group can be clearly attributed to fahlore minerals, whereas Pb-Zn can be related also to galena (PbS) and sphalerite (ZnS) sulphides which are less abundant. The correlation plot of Fig. 4 represents the Pearson correlation matrix with cluster analysis on log_10_ transformed data of all the trace elements analysed on waste rocks, soils, and sediments.

Two groups of elements are identifiable based on linear correlation and cluster analysis of major and trace components. The “metallogenic component” consists of the chalcophile elements: Cu-Sb-Hg-Ag-Zn-Pb-As-Cd which are metals and metalloids associated with mineralisation. In contrast, the “lithogenic component” is constituted by elements such as Mo-Fe-Cr-Tl-Ge-Mn, associated with the host rocks and adjacent lithologies such as limestones, metasediments, and red sandstones (Fig. 1). Cluster analysis confirms tight relationships among Cu, Sb, Ag, and Zn thus reflecting the abundant distribution of (Hg, Zn)-rich tetrahedrite. Conversely, the Pb-As-Cd association might be related to As-Cd rich galena as yet unreported mineral phases. Zinc was included within the metallogenic component, but its occurrence as a common minor constituent of the upper continental crust (Rudnick and Gao 2003) placed it between the metallogenic and lithogenic components. Among the second group of elements, the most correlated are Cr, Fe, Tl, and Ge, which unlikely were present in the ore-bearing minerals. Interestingly, Tl and Ge behave like siderophile elements in the Mt. Avanza mining site although they can be usually found as chalcophile elements in many sulphide ore deposits (Barago et al. 2021; Leach et al. 2010; Pavoni et al. 2017). In fact, since Tl has ionic radius and charge (+1) similar to the alkali metals such as potassium (K), it is reasonably hosted in K-bearing mica minerals of the Carboniferous/Ordovician mica schist formation (Peter and Viraraghavan 2005), whereas most of Ge is reasonably dispersed through silicate minerals due to the substitution of Ge^4+^ with the geochemically similar Si^4+^ (Rosenberg 2009).

**Fig. 5:**
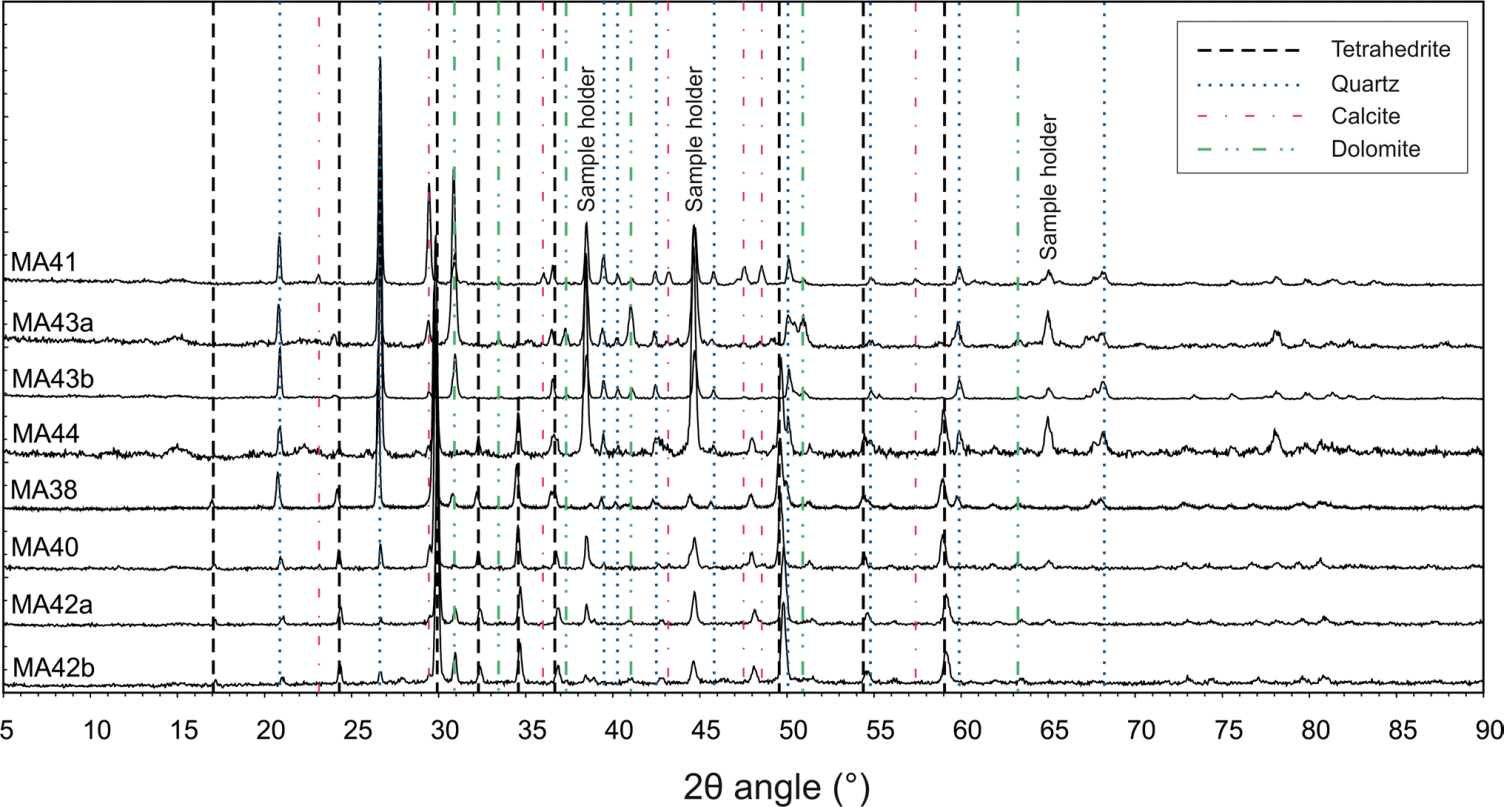
X-ray powder diffractograms (XRD) of the manually separated minerals

Four mineral phases have clearly been identified via XRD powder diffraction performed on manually separated specimens (*n* = 8): tetrahedrite, dolomite, calcite, and quartz. The patterns are presented in Fig. 5. In tetrahedrite, there could be several substitutions of cations including Hg, Ag, Zn, and Fe. All these substitutions affected the cell edges (a) of the tetrahedrite according to the general formula by Johnson et al. (1987):

$$\text{a}\;({\overset{\circ}{\text{A}}})=10.379+0.082\left(\text{Ag}\right)-0.01\left(\text{Ag}^{2}\right)-0.009\left(\text{Cu}^{\ast}\right)+0.066\left(\text{Hg}\right)-0.038\left(\text{As}\right)+0.144\left(\text{Bi}\right)$$

where Cu* = 2.0 – (Fe + Zn + Hg + Cd) and the coefficient of the Hg term was corrected according to Di Benedetto et al. (2002). Hall (1972) found a cell edge equal to 10.3191 ± 0.0005 Å for pure synthetic tetrahedrite. Biagioni et al. (2020a) found a cell edge value of 10.4725 ± 0.0001 to 10.5057 ± 0.0008 Å for specimens of tetrahedrite-(Hg) with a chemical content of Hg in the range 15–20 wt.%. Biagioni et al. (2020b) found a value of 10.3798 ± 0.0008 Å for a tetrahedrite-(Zn). Considering the chemical analyses of tetrahedrite from Mt. Avanza (Casari et al. 1996) where the average Hg content was 4.26 wt.%, and Zn was about 5.62 wt.%, the cell edge found here equal to 10.3929 ± 0.0001 Å confirmed these data being higher than that of tetrahedrite-(Zn) and in the middle between that of the synthetic tetrahedrite and the tetrahedrite-(Hg) specimens. Quartz, calcite and dolomite were identified in variable amounts and were gangue constituents as indicated by Feruglio (1966).

### Sediment quality

**Fig. 6:**
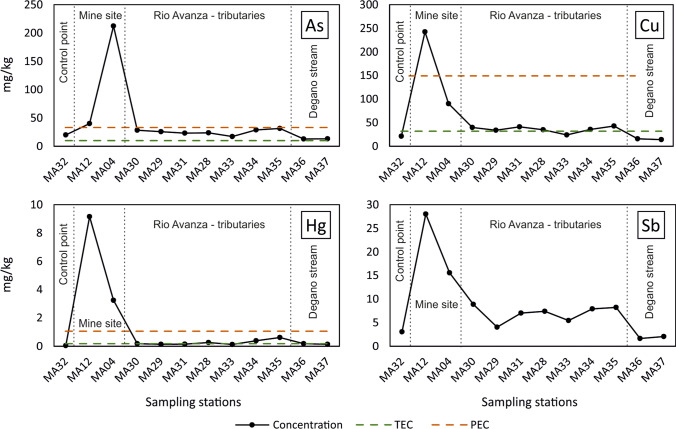
Concentrations of As, Cu, Hg, and Sb of stream sediments collected at the historic mining district of Mt. Avanza. Threshold effect concentration (TEC) and probable effect concentrations (PEC) from MacDonald et al. (2000)

With respect to sediments, the variability of metal(loid) concentrations was strictly related to the distance from the mining district, decreasing “exponentially” moving away from the mine (Fig. 6). Maximum concentrations were 212 mg/kg As (MA04) and 242 mg/kg Cu, 9.15 mg/kg Hg and 28.0 mg/kg Sb for MA12 sample (Tab. S1) as a consequence of the extraction activity and transport of sediments from the mine to the external area.

The contamination assessment was provided by means of the geoaccumulation index (I_geo_) (Müller 1969), calculated according to the following equation:

$${I}_{geo}={{\log}}_2\left(\frac{C_n}{1.5{B}_n}\right)$$

*C*_n_ measured element concentration; *B*_n_ background element concentration

The MA32 sample was chosen as the control point (Garcia-Ordiales et al. 2017) and B_n_, since it was collected in the Rio Avanza upstream the confluence of the tributaries (Fig. 2). The I_geo_ values (Table 3) between 2 and 3, indicating moderately contaminated sediments, were found for As, Cu, and Sb in the mine area. However, sediments extremely contaminated by Hg (I_geo_ > 5) were found in the mine area, as well as moderately to heavily contaminated sediments (I_geo_ = 2–3) were found in the Rio Avanza stream. Downstream sediments (e.g. MA37), far from the source, are characterised by lower I_geo_ values, thus indicating that the environmental impact is restricted to the area surrounding the mine site, whereas it is very low on the drainage basin. It is reasonable to think that the volumes extracted and processed were relatively small and decades of flushing have diluted element concentrations in the stream sediments. In fact, the overall legacy impact of the mining activity at Mt. Avanza is very low compared to other areas where hundreds or thousands of milligram per kilogram of PTEs in stream sediments were also found at tens of kilometres from large and historical mine sites (Müller et al. 1994, Covelli et al., 2001; Garcia-Ordiales et al. 2017).

**Table 3:**
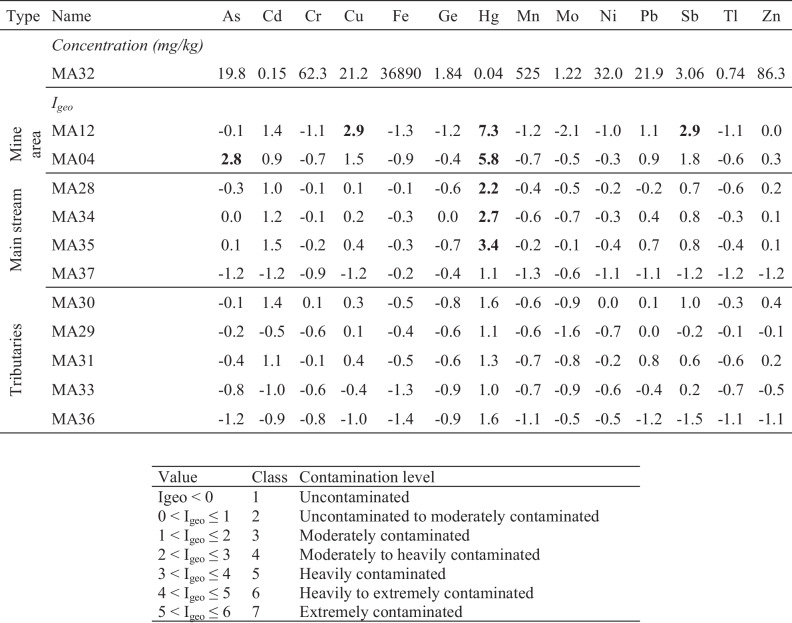
Geoaccumulation index (Igeo) (Müller 1969) for stream sediments of the Rio del Lago-Slizza stream sediments. Igeo > 5 are highlighted in bold. MA32 was selected as “control sample”.

PTE concentrations in sediments were compared with sediment quality guidelines (SQG) for metal toxicity in freshwater ecosystems (Fig. 6). TEC (threshold effect concentration) and PEC (probable effect concentration) are used to identify the concentration ranges of chemicals associated with sediment toxicity and biological effects in sediment-dwelling organisms (MacDonald et al. 2000). Moreover, they provide a preliminary basis for assessing sediment quality conditions in freshwater ecosystems, being PEC correlated to the incidence of toxicity. Our data indicate that in the mining area, As, Cu and Hg concentrations are almost always above the PEC value, attesting that a probable adverse effect may frequently occur, whereas most of the stream sediments from the Rio Avanza stream are between TEC and PEC, thus suggesting that adverse effects may occasionally occur. Speciation analyses on sediment samples, especially considering the bioavailable fraction, could be of help in depicting the real impact on the aquatic biota in the drainage basin affected by the past mining activities.

### Hydrogeochemistry and transport of dissolved metal(loid)s

The investigated water samples showed overall near-neutral to slightly alkaline conditions (7.52–8.78; Table S2). Average pH value of 8.0 ± 0.4 was found in the mine drainage water, whereas surface water collected from the Rio Avanza stream and its secondary tributaries showed slightly more alkaline conditions (pH = 8.4 ± 0.1). This indicates the natural pH buffering capacities following dissolution of limestones, which is one of the main lithologies outcropping in the investigated area, as also confirmed by HCO_3_^-^ concentrations ranging overall between 132 and 221 mg/L.

Similar to other decommissioned mining districts characterised by carbonate host rock (Hiller et al. 2013; Petrini et al. 2016; Pavoni et al. 2018), the waters draining the decommissioned mining district of Mt. Avanza belong to neutral mine drainage (NMD) and no evidence of acid mine drainage (AMD) was observed, although AMD is quite commonly observed in areas affected by the mining of sulphides (Aguilar-Carrillo et al. 2022; D’Orazio et al. 2017; De Giudici et al. 2019; Li et al. 2020; Perotti et al. 2018; Zhou et al. 2017). Among the different sampling campaigns, the mine drainage water showed relatively constant EC (175 ± 40 μS cm^-1^) and TDS (92.5 ± 18.8 mg/L) values, which were found to be lower with respect to those observed in the Rio Avanza stream water samples (EC = 538 ± 223 μS/cm, TDS = 304 ± 93 mg/L) most likely due to the dissolution of gypsum lithologies and resuspension events related to maintenance and up-keep on the slopes upstream from the sampling stations along the Rio Avanza stream basin. Oxidative conditions (mean 270 ± 70 mV) and relatively elevated concentrations of dissolved oxygen (mean 7.4 ± 0.5 mg/L) were observed in all the investigated water samples.

**Fig. 7:**
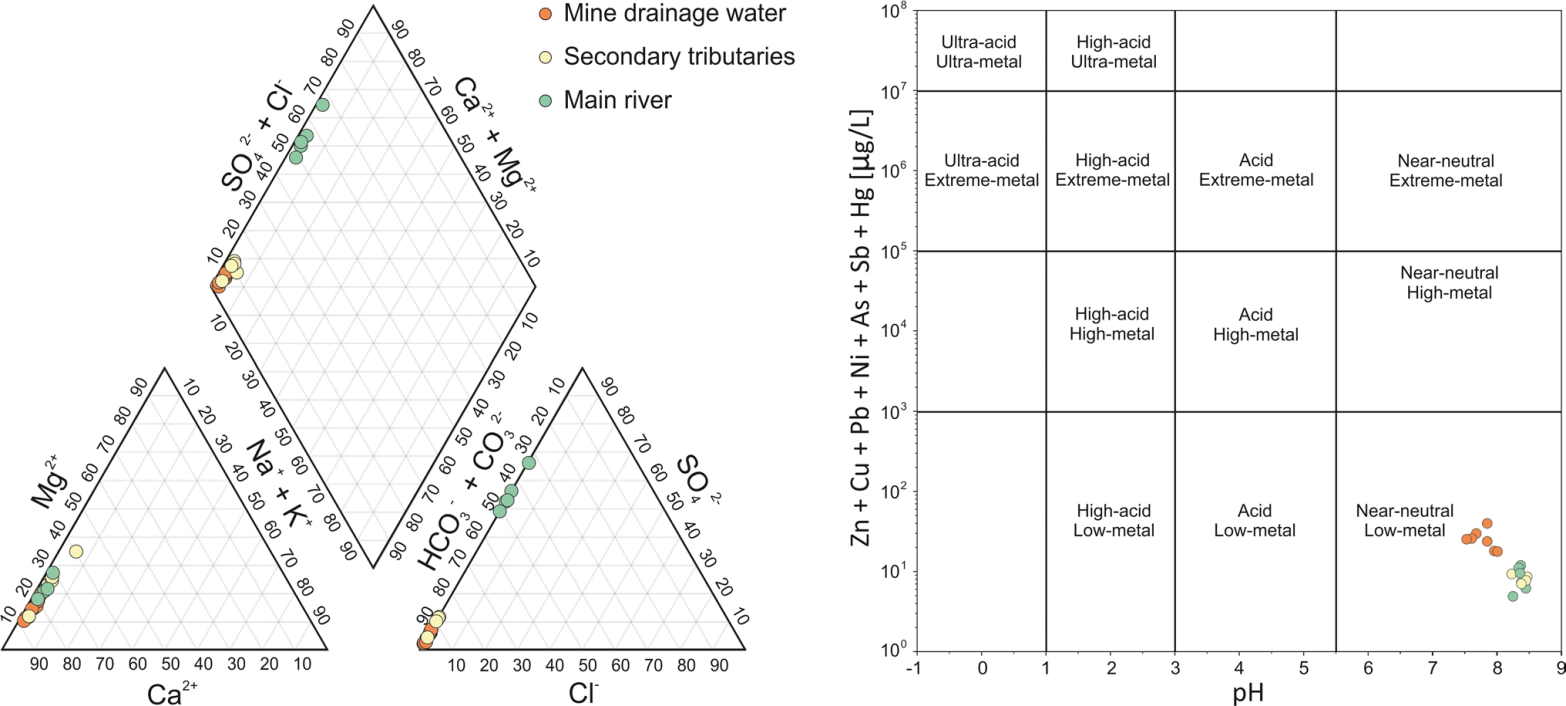
Piper (left) and Ficklin (right) diagrams of the different groups of water collected at the decommissioned mining district of Mt. Avanza

According to the Piper diagram (Piper 1944), the waters mostly belong to the calcium-bicarbonate hydrochemical facies as the result of carbonate rock dissolution (Fig. 7, left). Conversely, calcium-sulphate facies was identified as the dominant hydrochemical facies in the case of the Rio Avanza stream water, most likely due to weathering of Permian gypsum lithologies outcropping along the stream basin (Fig. 2). Indeed, the SO_4_^2-^ concentration was found to be notably higher in the Rio Avanza main stream (mean 198 ± 71 mg/L) rather than in its secondary tributaries (mean 13.6 ± 4.6 mg/L) as well as in the mine drainage water (mean 6.7 ± 2.9 mg/L). Moreover, the content of SO_4_^2-^ still remained elevated in the Degano stream water collected downstream from the confluence with the Rio Avanza stream (196 mg/L at site MA37) and notably higher than that observed upstream (14.2 mg/L at site MA36).

The relatively low concentration of SO_4_^2-^ in the mine drainage water suggests that sulphide oxidation did not significantly contribute, although quite a significant correlation was observed between SO_4_^2-^ and dissolved PTEs potentially released following sulphide oxidation in the mine drainage water, excluding site MA04 (Sb, *r* = 0.903, *N* = 8, *p* < 0.01; As, *r* = 0.828, *N* = 8, *p* < 0.01; Cu, *r* = 0.639, *N* = 8, *p* < 0.05)

In keeping with the Ficklin diagram (Plumlee et al. 1999), all the investigated water samples were classified as “near-neutral, low-metal” water (Fig. 7, right) thus confirming (1) the role of carbonate dissolution which resulted in natural pH buffering effects, (2) the moderate stability of primary and secondary ore-bearing minerals, and (3) the limited extent of the impact of mining activities on water composition. However, stream water from the main stream and its secondary tributaries clearly differed from the mine drainage water samples which showed a slightly higher PTEs content in the Ficklin diagram, indicating that a geochemical signature from the Mt. Avanza mining district can be observed.

**Fig. 8:**
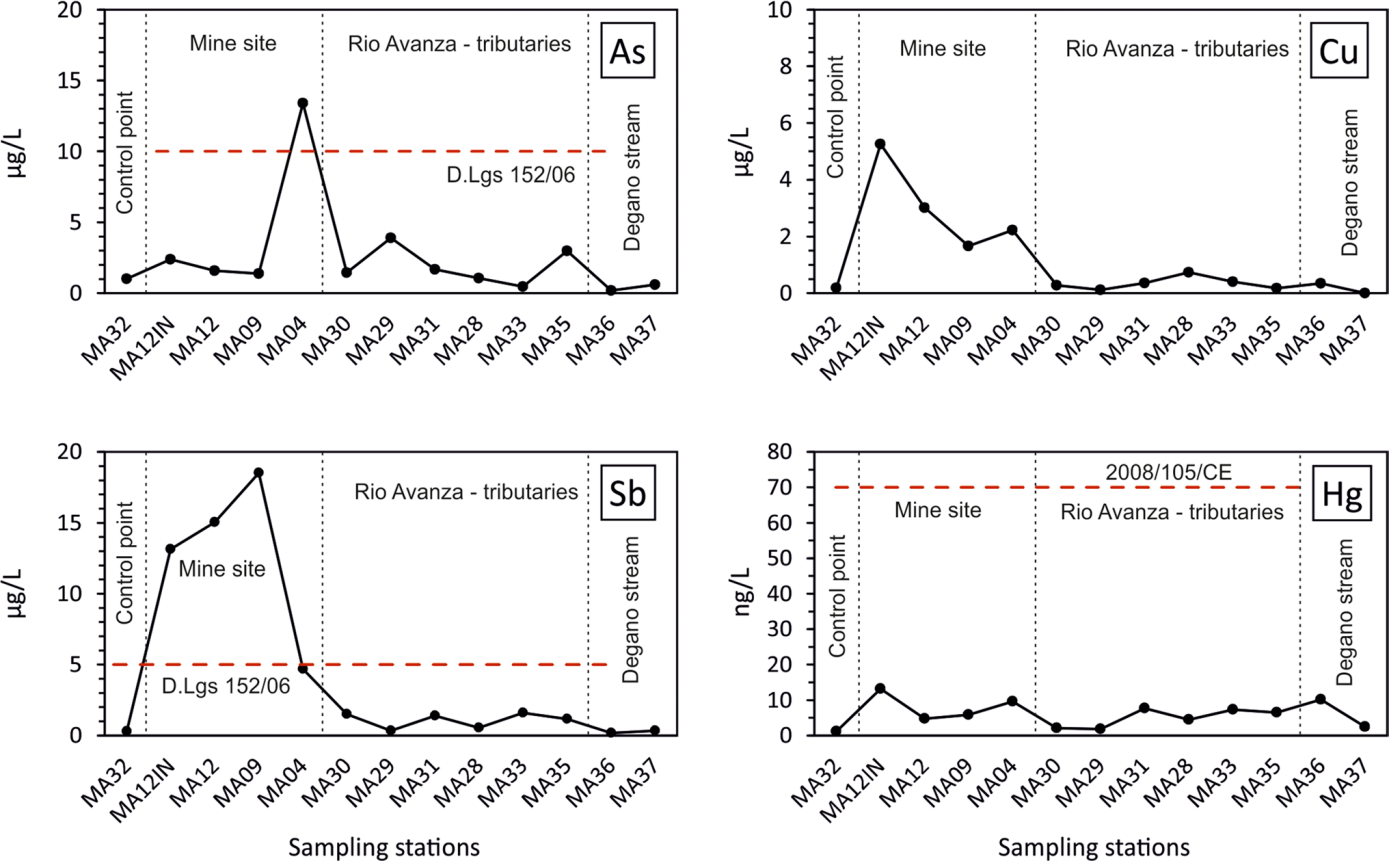
Concentrations of As, Cu, Hg, and Sb in water collected at the historic mining district of Mt. Avanza. D.Lgs. 152/06: Italian reference legislation for groundwater quality. 2008/105/CE European water quality standard

Regarding the occurrence of dissolved PTEs, relatively low concentrations were observed (Table S2) and a general dilution effect occurred by moving downstream from the mining district (Fig. 8). The same dilution effect was observed for sediments and was especially evident in the case of Cu, Sb, and Hg. In the case of water, the dilution of PTEs was notable only for Cu, Sb, and As which were more abundant in the mine drainage water samples (maximum concentrations: Cu 8.28 μg/L at sampling station MA12IN, Sb = 20.3 μg/L at sampling station MA09, and As = 14.8 μg/L at the sampling station MA04) thus suggesting water-rock interaction processes involving the main mineralisation at Mt. Avanza, and decreasing downstream (Fig. 8). Moreover, the Rio Avanza water collected upstream from the mining district showed concentrations of one order of magnitude lower (site MA32: Cu = 0.18 μg/L, Sb = 0.30 μg/L and As = 1.02 μg/L).

Conversely, dissolved Hg was found to be 3 orders of magnitude less abundant (ranging between 1.17 and 13.2 ng/L) and marked differences were not observed among all the water samples (Fig. 2), indicating that there was not a significant release of dissolved Hg in the water basin. Moreover, Hg concentrations were always below the European maximum allowable concentration of 70 ng/L in surface waters (European Directive 2008/105/CE). Comparatively, the neutral waters of the world-class cinnabar Idrija mining district can reach concentrations of dissolved Hg more than one order of magnitude higher (up to 359 ng/L; Baptista-Salazar et al. 2017). Although Hg is abundant in tetrahedrite minerals (Table 1) and in the analysed solid fraction (maximum concentration of 476 mg/kg at site MA05), this evidence suggests that Hg may be not easily released in solution following mineral weathering (Fu et al. 2010).

Contrary to what was expected as the result of tetrahedrite and tennantite oxidation resulting in the release of Sb and As in solution (Borčinová Radková et al. 2017; Hiller et al. 2013), As behaved differently in terms of mobility with respect to Sb under oxidising conditions (Hiller et al. 2012). Indeed, according to Majzlan et al. (2018), Sb is generally more mobile and easily released during weathering of tetrahedrite-rich mining waste than As (50% and 10% of release for Sb and As, respectively) which is commonly involved in attenuation processes as the result of adsorption/precipitation of Fe oxyhydroxides (HFO) (Casiot et al. 2007; Hiller et al. 2012). However, although HFO may play a crucial role in regulating the mobility of both As and Sb (Herath et al. 2017; Ritchie et al. 2013 and references therein), this did not appeared to be a dominant process in the investigated area where 1) a small amount of primary Fe sulphides were reported,and subsequently secondary HFOs are supposed to be scarce, and (2) absence of correlation between As-Sb and Fe in the solid matrix, whereas the latter showed slightly constant concentration in all the investigated stream sediments (3.68 ± 1.00 %). According to Craw et al. (2004), in the absence of attenuation processes mediated by HFOs, Sb may be readily leached from mine tailings and deposits and dispersed in the surrounding environment. In contrast, some natural attenuation process of Sb could act in the Mt. Avanza district. The formation of the so-called antimony ochre is related to Sb secondary minerals in the form of coatings which are a product of alteration from tetrahedrite minerals. Such supergene minerals could be constituted by oxide minerals from the Roméite group (A_2_B_2_X_6_Y formula in which Sb^5+^ predominated in the B-site) (Álvarez-Ayuso 2021; Lopes et al. 2021; Borčinová Radková et al. 2017; Bortolozzi et al. 2015). It is less likely but not impossible that the attenuation could be also helped by the formation of Fe antimonates such as tripuhyite (FeSbO_4_) (Berlepsch et al. 2003). Such attenuation processes could limit the release of Sb in water being interesting potential natural attenuation processes that should be more thoroughly investigated in the future.

**Fig. 9:**
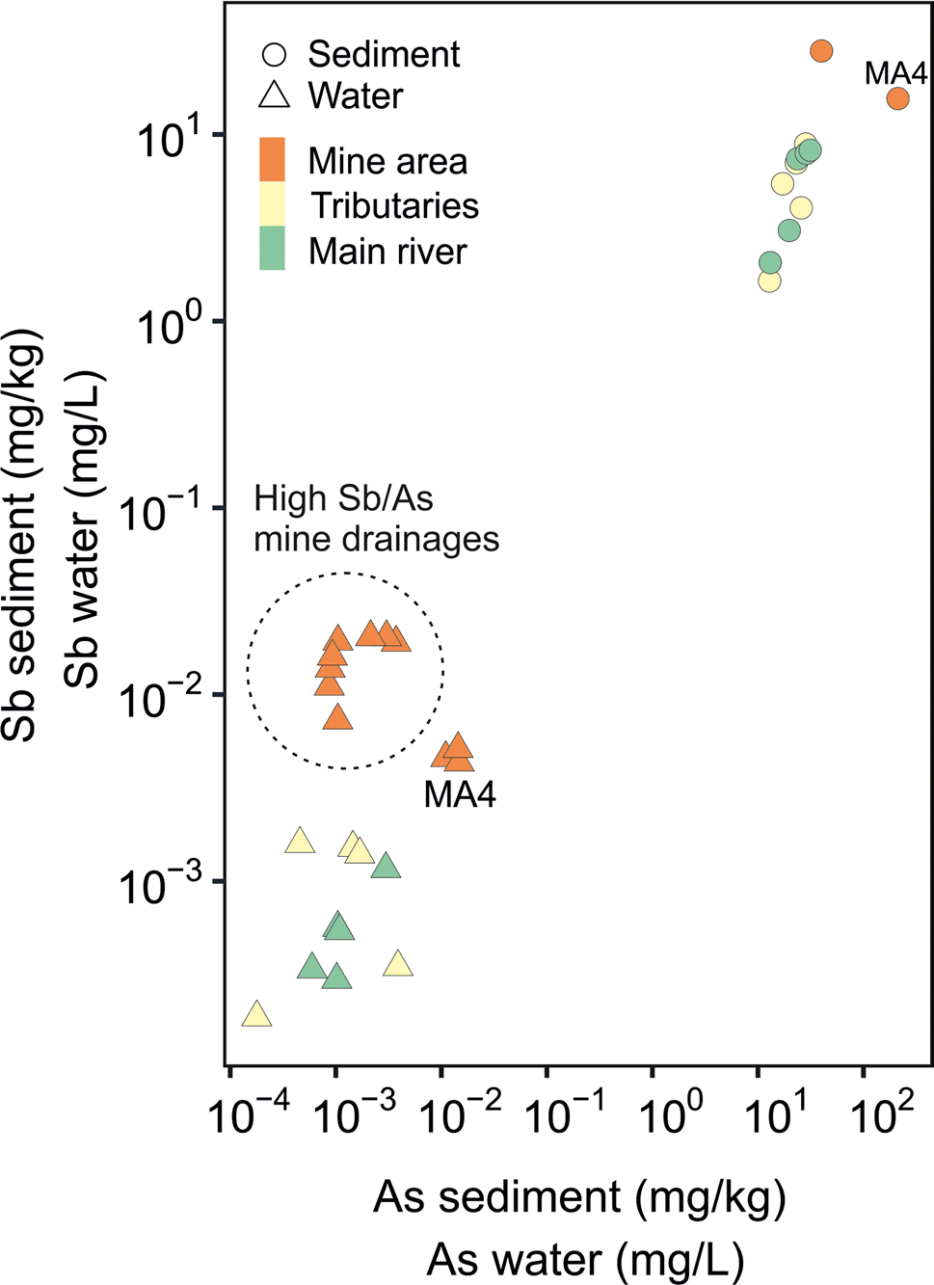
Correlation between Sb and As in water and sediments collected from the Mt. Avanza mining district

In this study, the Sb/As ratio in water clearly decreased by moving downstream from the mining district (5.09–18.3 at the mining district to values < 1 downstream) and the mine drainage water generally showed low As content (mean 1.42 ± 0.84 μg/L) as well as both stream water collected from the main stream (mean 1.35 ± 0.94 μg/L) and its secondary tributaries (1.53 ± 1.46 μg/L). Antimony (Sb) appeared to be more mobile in the aquatic environment since it can be more easily desorbed and released with respect to As from tetrahedrite-bearing rocks of the mine drainage system in most of the mine drainage waters (Fu et al. 2010; Hiller et al. 2012) (Fig. 9), most likely due to the fact that Sb adsorption notably decreases with increasing pH (Filella et al. 2002b; Leuz et al. 2006; Martínez-Lladó et al. 2008).

Regarding the general water quality, due to a lack of proper national guidelines concerning water draining mineral deposits, the results from this research may only be compared to the Italian regulatory threshold limits for contaminated groundwaters (Italian D.Lgs. 152/2006 according to EU Directive 2000/60/EC and 2008/105/CE) highlighting a persistent Sb or As exceeding of the limit values (5 and 10 μg/L, respectively) in almost all the mine drainage water (Fig. 8). This is especially evident in the case of Sb since non-contaminated water usually shows concentrations below 1 μg/L (Filella et al. 2002a).

### Gaseous elemental mercury (GEM) occurrence in the mining district

**Fig. 10:**
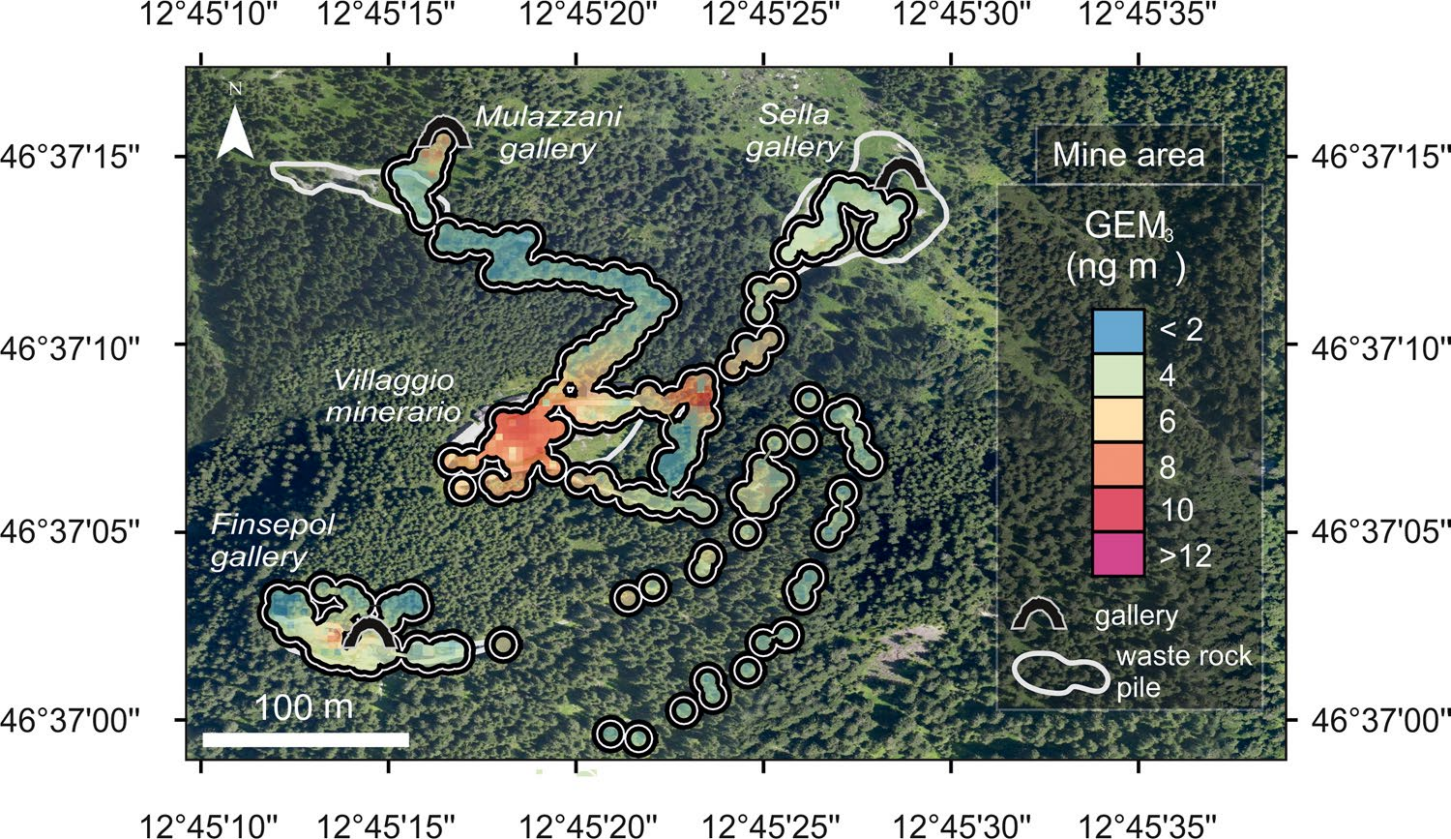
Detail of the spatial distribution of atmospheric GEM concentration at the Mt. Avanza mining site

The GEM concentrations recorded during the field survey within the Mt. Avanza mining site were relatively low, ranging between values below the instrumental LOD (2 ng/m^3^) up to a maximum of 25.4 ng/m^3^ (Table 2), with a mean GEM concentration of 4.78 ± 3.61 ng/m^3^. These values were slightly higher than the estimated natural background of atmospheric GEM for the Northern Hemisphere (1.5–1.7 ng/m^3^, Sprovieri et al. 2010) and not far from those recorded during a year-long monitoring at the remote site of Col Margherita, located ~75 km WSW from our study area in the North-Eastern Italian Alps (mean 3.14 ± 1.29 ng/m^3^; Vardè et al. 2022). Moreover, similar peak values of atmospheric GEM (up to 48.5 ng/m^3^) were reported for the other Hg impacted coastal, lagoonal areas and alluvial plains of the Friuli Venezia Giulia region (Acquavita et al., 2022; Barago et al., 2020; Floreani et al., 2020), which suffered extended contamination due to the transport by the Isonzo River of Hg-enriched material from the Idrija cinnabar mine, located ~100 km upstream these locations (Acquavita et al. 2022; Covelli et al. 2001, 2007). Overall, in the surroundings of the mining district, peaks of GEM concentrations higher than 15 ng/m^3^ were observed above the abandoned waste rock piles and along the roads where Hg-rich material was used for construction of the various facilities (Fig. 10).

The spatial distribution of GEM in the study area roughly corresponded to that of Hg concentrations in soils and waste rock piles (Fig. 3), supporting the hypothesis of emission from the Hg-rich substrate. As observed at other former Hg-mining sites, abandoned wastes and contaminated soils can represent relevant sources of this metal into the atmosphere even for decades after mining and metallurgical activities have ceased (e.g. Floreani et al., 2023; Higueras et al. 2013; Loredo et al. 2007; Nacht et al. 2004; Yan et al. 2019).

The highest mean GEM concentrations (9.47 ± 4.16 ng/m^3^) were indeed observed at the waste rock pile by “Villaggio Minerario” (Fig. 2b and Fig. 9), characterised by high Hg concentration in the substrate (up to 56.9 mg/kg). Moreover, these measurements were taken during the period of maximum irradiation, at midday, when the enhanced formation of GEM through photoreduction and a following evasion is expected (Dalziel and Tordon 2014; Fantozzi et al. 2013; Floreani et al., 2023; Kotnik et al. 2005) also considering the lack of vegetation cover on the waste rock piles (Loredo et al. 2007). As observed in other field studies (e.g. Coolbaugh et al. 2002; Floreani et al., 2023), vegetation significantly reduces GEM release from enriched substrates compared to bare areas: this effect can be attributed to the reduction of solar radiation reaching the surface, which decreases the GEM generation rate through photoreduction (Liu et al. 2014). Moreover, lower GEM emission under canopy shading may also be related to lower soil temperatures, which limit the diffusion of GEM to the atmosphere and can affect the rates of microbial reactions involved in Hg reduction (Mazur et al. 2014; Yuan et al. 2019).

Lower GEM values were found at a waste rock pile near the entrance of the “Sella” gallery, characterised by lower Hg concentration in the substrate (up to 13.3 mg/kg) possibly due to the burial of residues containing tetrahedrite, which reduced the amount of Hg in surface layers available for evasion in gaseous form. Remediation of Hg-rich wastes by covering with inert material, soil, and vegetation has proven to be effective at significantly reducing GEM concentrations in the air, as previously reported for the Almadén (Spain) and Tongren (China) Hg-mining districts (Higueras et al. 2013; Yan et al. 2019).

Slight increases in GEM concentrations from background levels (up to ~10 ng/m^3^) were observed near the entrances of the “Finsepol” and “Mulazzani” mine galleries, likely due to the occurrence of Hg in mineral phases similar to that observed by Higueras et al. (2012) in the Potosì mining area (Bolivia), where the occurrence of tetrahedrite was reported. The values of GEM in the air observed in that area were similar to those found in this study at Mt. Avanza, although concentrations from hundreds to thousands of ng/m^3^ are frequently reported in sites characterised by notably elevated Hg contents in substrates, such as near larger former Hg mines and cinnabar roasting plants (e.g. Ao et al. 2017; Fornasaro et al. 2022; Higueras et al. 2013; Kocman et al. 2011).

Far from the above-described point sources, GEM concentrations dropped to values similar to the natural background within distances of ~100 m, likely due to efficient atmospheric dilution in the mixing layer under the sunny weather conditions which occurred during sampling (Cabassi et al. 2017). Similar spatial patterns characterised by sharp decreases in GEM within few hundreds of meters from the emission source, independently from its absolute importance, were also observed at world-class Hg-mining districts such as Almadén (Esbrí et al. 2020, Llanos et al. 2010) and Mt. Amiata (Vaselli et al. 2013) confirming the importance of atmospheric dilution processes in regulating GEM dispersion. Indeed, GEM concentration observed in nearby Forni Avoltri and Pierabech rural areas, were relatively low, (mean 2.20 ± 0.76 ng/m^3^, max = 4.50 ng/m^3^, Table 2) even though in the past such villages were the sites of roasting plants and furnaces. Moreover, as forest ecosystems can serve as net sinks for atmospheric Hg (Yuan et al. 2019), GEM spatial spreading could also be limited through canopy scavenging by local coniferous vegetation thanks to stomatal uptake and depositions through litterfall and throughfall (Wright et al. 2016). Several studies reported higher total Hg depositions in coniferous rather than deciduous forests (Blackwell and Driscoll 2015; Demers et al. 2007; Witt et al. 2009): this, coupled with reduced emissions from the forest floor generally observed in these ecosystems (Agnan et al. 2016), can result in the notable accumulation of Hg in soils (Blackwell and Driscoll 2015; Richardson and Friedland 2015). Further research is needed to assess GEM levels, especially in summer, under conditions of high irradiation and temperatures more conducive for Hg release and thus to quantify the role of the gaseous exchanges in controlling the atmospheric Hg pool at the Mt. Avanza district.

## Conclusions

The geochemical signature of the past mining activity at the fahlore Cu-Sb(-Ag) Mt. Avanza ore deposit was evidenced by notable concentrations of the elements associated with the (Hg-Zn)-rich tetrahedrite in mine wastes, soils, and stream sediments (max: Cu = 4019 mg/kg, Sb = 1049 mg/kg, Pb = 1216 mg/kg, Zn = 1204 mg/kg, As = 654 mg/kg and Hg = 473 mg/kg). Results from this research suggest that extraction activities as well as the by-products employed in the constructions of roads and mine facilities contribute to a heterogeneous distribution of PTE concentrations in the soil matrix. Conversely, notable amounts of PTEs in the stream sediments appeared to be restricted to the mining area as shown by the general decrease in the PTE concentrations with increasing distance from the source area. This was confirmed by the I_geo_ index values which were found to be significantly high inside the mine area. Interestingly, Tl and Ge are associated with the “lithogenic components” substituting K and Si in silicate minerals, respectively, and are not associated to the ore-bearing minerals like in many other base metal ore deposits (e.g. carbonate-hosted Pb-Zn ores).

Regarding solid-water interactions, the water draining the mining district belonged to a “neutral mine drainage” (NMD) as the result of pH buffering effects due to dissolution of the Devonian carbonate host rocks, promoting the release of Sb, which was found to be generally more mobile than As in solution. Although mine drainages often exceeded the national regulatory limits for Sb and As, relatively low dissolved concentrations of the main metal(loid)s were observed, suggesting the moderate stability of the tetrahedrite and other minor ore-bearing minerals as well as the occurrence of natural attenuation processes along the river system, most likely due to dilution, (co-)precipitation, and potential sorption processes. Therefore, it appears that the Mt. Avanza mine is not a relevant source of contamination for the area.

Surprisingly, the geochemical behaviour of Hg in the fahlore site appeared similar to cinnabar deposits investigated around the world. Indeed, dissolved Hg concentrations were generally very low compared to other metal(loid)s such as As, Cu, and Sb, testifying to the weak solubility of this element which remained partitioned in the crystalline structures. However, similar to other cinnabar mining sites, the Hg-rich ore-bearing substrate was identified as a potential source of Hg into the atmosphere and real-time measurements of GEM were confirmed as a suitable preliminary tool to identify mine wastes and Hg-contaminated solid environmental matrices.

## Supplementary Information

ESM 1
