# Supplementary material for: Environmental impact of potentially toxic elements on soils, sediments, waters, and air nearby an abandoned Hg-rich fahlore mine (Mt. Avanza, Carnic Alps, NE Italy)
Source: Environ Sci Pollut Res Int. 2023 Apr 14;30(23):63754–75. doi: 10.1007/s11356-023-26629-7 (PMC10172279; doi:10.1007/s11356-023-26629-7)
Supplement: Supplementary file 1 — ESM 1 [file 11356_2023_26629_MOESM1_ESM.docx]

Environmental impact of potentially toxic elements on soils, sediments, waters and air nearby an abandoned Hg-rich fahlore mine (Mt. Avanza, Carnic Alps, NE Italy)

Environmental Science and Pollution Research

Nicolò Barago^1,*^, Cristiano Mastroianni^1^, Elena Pavoni^1^, Federico Floreani^1,2^, Filippo Parisi^1^, Davide Lenaz^1^, Stefano Covelli^1^

^1^ Dipartimento di Matematica e Geoscienze, Università di Trieste, Via Weiss 2, 34128, Trieste, Italy

^2^ Dipartimento di Scienze della Vita, Università di Trieste, Via Licio Giorgieri 5, 34127, Trieste, Italy

* Corresponding author.

*E-mail address:* nicolo.barago@phd.units.it (N. Barago)

**Supplementary Material**

**Table S1** Minimum and maximum values of concentration of the main major and trace elements of mine waste, soil samples in the Mt. Avanza mine area, and sediments sampled in the mine area and downstream in the Avanza-Degano valley.

| **Parameter** | **Unit** | **Waste rock pile (n = 6)** | **Soil (n = 18)** | **Sediment (n = 12)** |
| --- | --- | --- | --- | --- |
| Ag | mg/kg | 0.15 - 14.1 | <LOD - 5.54 | <LOD - 0.65 |
| As | mg/kg | 8.20 - 654 | 12.1 - 162 | 12.9 - 212 |
| Cd | mg/kg | 0.17 - 6.19 | 0.06 - 2.03 | 0.10 - 0.65 |
| Cr | mg/kg | 6.91 - 88.0 | 25.0 - 95.1 | 49.0 - 102 |
| Cu | mg/kg | 31.3 - 4019 | 10.13 - 1556.8 | 13.97 - 242 |
| Ge | mg/kg | 0.20 - 2.26 | 0.84 - 1.75 | 1.20 - 2.83 |
| Fe | mg/kg | 3248 - 36895 | 8042 - 39296 | 20816 - 49918 |
| Hg | mg/kg | 1.42 - 473 | 0.21 - 132 | 0.04 - 9.15 |
| Mn | mg/kg | 138 - 689 | 185 - 1227 | 247 - 667 |
| Mo | mg/kg | <LOD - 1.33 | 0.44 - 1.45 | 0.59 - 1.69 |
| Pb | mg/kg | 19.4 - 397 | 26.4 - 1216 | 14.7 - 78.8 |
| Sn | mg/kg | <LOD - 3.92 | <LOD - 3.71 | <LOD |
| Sb | mg/kg | 5.00 - 1049 | 2.74 - 153 | 1.64 - 28.0 |
| Tl | mg/kg | 0.09 - 1.07 | 0.31 - 0.95 | 0.50 - 1.04 |
| Zn | mg/kg | 20.3 - 553 | 62.0 - 309 | 57.1 - 171 |

**Table S2** Minimum and maximum values of physico-chemical parameters (pH, T, ORP, EC, TDS, DO), major ions (Ca^2+^, Mg^2+^, Na^+^, K^+^, F^-^, Cl^-^, NO_3_^-^, SO_4_^2-^, CO_3_^2-^, HCO_3_^-^) and trace elements (Ba, As, Cu, Fe, Mn, Mo, Pb, Zn, Sb, Hg) in mine drainage waters, Rio Avanza stream water and other surface water representative of minor tributaries and the Degano stream, sampled upstream and downstream its confluence with the Rio Avanza stream.

| **Parameter** | **Unit** | **Mine drainage water (n = 11)** | **Main river (n = 5)** | **Secondary tributaries (n = 5)** |
| --- | --- | --- | --- | --- |
| pH |  | 7.52 - 8.78 | 8.24 - 8.44 | 8.22 - 8.46 |
| T | °C | 4.96 - 6.50 | 5.66 - 7.68 | 6.34 - 8.18 |
| ORP | mV | 74.0 - 303 | 72.0 - 301 | 287 - 294 |
| EC | μS cm^-1^ | 114 - 227 | 262 - 864 | 176 - 226 |
| TDS | mg/L | 54.0 - 113 | 213 - 432 | 88.0 - 113 |
| DO | mg/L | 5.61 - 12.3 | 6.64 - 8.20 | 6.67 - 8.08 |
| Ca^2+^ | mg/L | 36.9 - 42.4 | 82.3 - 197 | 34.1 - 42.8 |
| Mg^2+^ | mg/L | 2.65 - 6.49 | 14.0 - 26.1 | 3.25 - 11.7 |
| Na^+^ | mg/L | 0.49 - 1.07 | 2.85 - 4.65 | 0.85 - 2.68 |
| K^+^ | mg/L | 0.42 - 0.66 | 0.99 - 1.17 | 0.46 - 1.26 |
| F^-^ | mg/L | 0.01 - 0.10 | 0.02 - 0.29 | 0.03 - 0.05 |
| Cl^-^ | mg/L | 0.13 - 0.65 | 0.46 - 2.15 | 0.22 - 0.37 |
| NO_3_^-^ | mg/L | 0.37 - 1.25 | 2.01 - 2.97 | 1.04 - 4.29 |
| SO_4_^2-^ | mg/L | 2.44 - 10.2 | 124 - 316 | 5.40 - 16.2 |
| CO_3_^2-^ | mg/L | <LOD | <LOD - 10.8 | <LOD - 12.0 |
| HCO_3_^-^ | mg/L | 148 - 171 | 143 - 221 | 132 - 178 |
| Ba | mg/L | 0.04 - 0.10 | 0.05 - 0.10 | <LOD - 0.28 |
| As | μg/L | 0.87 - 14.8 | 0.60 - 2.98 | 0.18 - 3.89 |
| Cu | μg/L | 0.29 - 8.28 | <LOD - 1.08 | 0.12 - 0.41 |
| Fe | μg/L | 4.16 - 14.1 | 10.5 - 27.5 | 3.66 - 5.42 |
| Mn | μg/L | 0.11 - 2.78 | 0.27 - 1.62 | 0.15 - 0.37 |
| Mo | μg/L | <LOD - 0.47 | 0.58 - 6.70 | <LOD - 0.96 |
| Pb | μg/L | <LOD - 1.02 | <LOD - 0.72 | <LOD |
| Zn | μg/L | <LOD - 11.0 | <LOD - 4.30 | <LOD - 1.90 |
| Sb | μg/L | 4.33 - 20.3 | 0.30 - 1.16 | 0.19 - 1.59 |
| Hg | ng/L | 2.41 - 13.2 | 1.17 - 6.49 | 1.81 - 10.2 |
